# Instrumented cervical fusion using patient specific end-plate conforming interbody devices with a micro-porous structure in nine dogs with disk-associated cervical spondylomyelopathy

**DOI:** 10.3389/fvets.2023.1208593

**Published:** 2023-06-26

**Authors:** Colin J. Driver, Victor Lopez, Ben Walton, Dan Jones, Rory Fentem, Andrew Tomlinson, Jeremy Rose

**Affiliations:** ^1^Lumbry Park Veterinary Specialists, CVS Referrals, Alton, United Kingdom; ^2^School of Engineering, University of Liverpool, Liverpool, United Kingdom; ^3^Fusion Implants, Liverpool, United Kingdom; ^4^Movement Referrals, Preston Brook, United Kingdom; ^5^Small Animal Teaching Hospital, University of Liverpool, Neston, United Kingdom

**Keywords:** cervical spondylomyelopathy, wobbler syndrome, vertebral distraction-fusion, custom implants, veterinary spinal surgery

## Abstract

**Objective:**

To report the medium and long-term outcome of nine dogs with disk-associated cervical spondylomyelopathy (DA-CSM), treated by instrumented interbody fusion using patient specific end-plate conforming device that features a micro-porous structure to facilitate bone in-growth.

**Study design:**

A retrospective clinical study.

**Animals:**

Nine medium and large breed dogs.

**Methods:**

Medical records at two institutions were reviewed between January 2020 and 2023. Following magnetic resonance imaging (MRI) diagnosis of DA-CSM, pre-operative computed tomography (CT) scans were exported to computer software for *in-silico* surgical planning. Interbody devices were 3D-manufactured by selecting laser melting in titanium alloy. These were surgically implanted at 13 segments alongside mono-or bi-cortical vertebral stabilization systems. Follow-up included neurologic scoring and CT scans post-operative, at medium-term follow up and at long-term follow-up where possible. Interbody fusion and implant subsidence were evaluated from follow-up CT scans.

**Results:**

Nine dogs were diagnosed with DA-CSM between C5-C7 at a total of 13 operated segments. Medium-term follow up was obtained between 2 and 8 months post-operative (3.00 ± 1.82 months). Neurologic scoring improved (*p* = 0.009) in eight of nine dogs. Distraction was significant (*p* < 0.001) at all segments. Fusion was evident at 12/13 segments. Subsidence was evident at 3/13 operated segments but was only considered clinically relevant in one dog that did not improve; as clinical signs were mild, revision surgery was not recommended. Long-term follow up was obtained between 9 and 33 months (14.23 ± 8.24 months); improvement was sustained in 8 dogs. The dog that suffered worsened thoracic limb paresis at medium-term follow up was also diagnosed with immune-mediated polyarthropathy (IMPA) and was euthanased 9 months post-operative due to unacceptable side-effects of corticosteroid therapy.

**Conclusion:**

End-plate conforming interbody devices with a micro-porous structure were designed, manufactured, and successfully implanted in dog with DA-CSM. This resulted in CT-determined fusion with minimal subsidence in the majority of operated segments.

**Clinical significance:**

The technique described can be used to distract and fuse cervical vertebrae in dogs with DA-CSM, with favorable medium-and long-term outcomes.

## 1. Introduction

Disk-associated cervical spondylomyelopathy (DA-CSM) is a degenerative disorder of the intervertebral disks, articular facet joints and supporting ligaments, which causes a compressive myelopathy and/or radiculopathy in medium to large breed dogs (1–4). Compression can occur in a static fashion, or through movement; with compression more evident when imaging the vertebral column in extension or flexion and potentially alleviated when providing vertebral distraction (5–9).

The disorder is typically expected to result in progressive clinical signs and surgical management appears to be of benefit in comparison to medical treatment (1, 9–17). The objective of surgery is to directly or indirectly decompress the spinal cord and nerve roots. Proposed surgical techniques can be categorized as decompressive only (to directly decompress from a ventral or dorsal approach), indirect decompression without motion preservation (vertebral distraction with stabilization or fusion) and indirect decompression with motion preservation (total disk arthroplasty). Success rates vary considerably in the literature (17–35). Immediate post-operative neurologic deterioration is a significant disadvantage of dorsal decompressive techniques (36).

Restoration of vertebral canal height and indirect cord decompression with the implantation of an interbody cage is a common surgical option for cervical spondylitic myelopathy in man. Achieving vertebral interbody fusion is desirable to limit the risk of vertebral instability and recurrence of compression (37, 38). Fusion can be aided by combining interbody spacers with plate fixation (38, 39). Similar techniques to attempt interbody fusion in dogs with DA-CSM include the use of allogenic cortical bone grafts, cement plugs and metallic spacers or devices such as screws, cages or other custom devices (19–35).

Alteration to the biomechanics of the stabilized segment can lead to adjacent segment pathology and disease (1, 24, 25, 28, 40, 41). Failure to achieve interbody fusion has been associated with implant failure or subsidence of interbody devices, causing narrowing of the intervertebral disk space and reoccurrence of spinal cord compression, with associated clinical signs (24, 25, 28, 41). Interbody cages that perfectly conform with adjacent vertebral end-plates present improved biomechanical performance compared to generic interbody cages in humans with significantly lower interface stress in all directions of motion, higher stability in flexion/extension, and minimal implant subsidence (42). The use of conforming devices in two dogs was recently described without the use of a micro-porous structure (32).

The objective of this retrospective study was to describe and evaluate the use of patient specific end-plate conforming interbody distraction fusion devices with a porous structure in combination with vertebral stabilization, in dogs with DA-CSM. Outcomes were assessed from long-term follow-up and included CT-based determination of vertebral fusion, implant subsidence and adjacent segment pathology.

## 2. Materials and methods

### 2.1. Animals

The medical records of two institutions were retrospectively reviewed between January 2020 and 2023, for dogs with clinical signs and MRI findings compatible with DA-CSM, that were treated by vertebral distraction-fusion using patient specific interbody devices. Ethical approval for the study was obtained from the main institutions internal review committee (CVS-2022-010). Data retrieved included patient signalment, clinical signs, diagnostic work-up, surgical technique and outcomes.

Neurologic examinations were scored according to the Texas Spinal Cord injury score (TSCIS) (43). Assessments and scoring were conducted by a board-certified neurologist or surgeon pre-operatively, post-operatively prior to discharge and at medium term (a minimum of 2 months) follow-up. Long-term follow-up, defined as a minimum period of 9 months post-operative (33), was retrospectively determined from physical examination, video analysis or pet owner telephone interview. Long-term outcome was categorized as deteriorated, stable, or improved.

### 2.2. Pre-operative imaging

The definitive diagnosis of DA-CSM was confirmed in all cases by high-field MRI (Philips Ingenia CV, 1.5 T, or Siemens Magnetom Essenza, 1.5 T) according to published criteria (1, 2, 10). Kinematic and distraction imaging was not performed in the study subjects. Immediately after disease confirmation, patients were transferred to CT (Toshiba Aquilion Prime, 80 slice, or Siemens Somatom Scope, 16 slice) and were positioned in dorsal recumbency within a trough, and with the cervical spine extended such that the angle of the mandible to the sternum was an angle approximately 30° to horizontal. This position was checked by the primary surgeon and documented using digital photography for later surgical reference. CT scans were obtained in a bone algorithm with 3D reconstruction for *in-silico* planning.

### 2.3. Device design and manufacture

**Figure 1:**
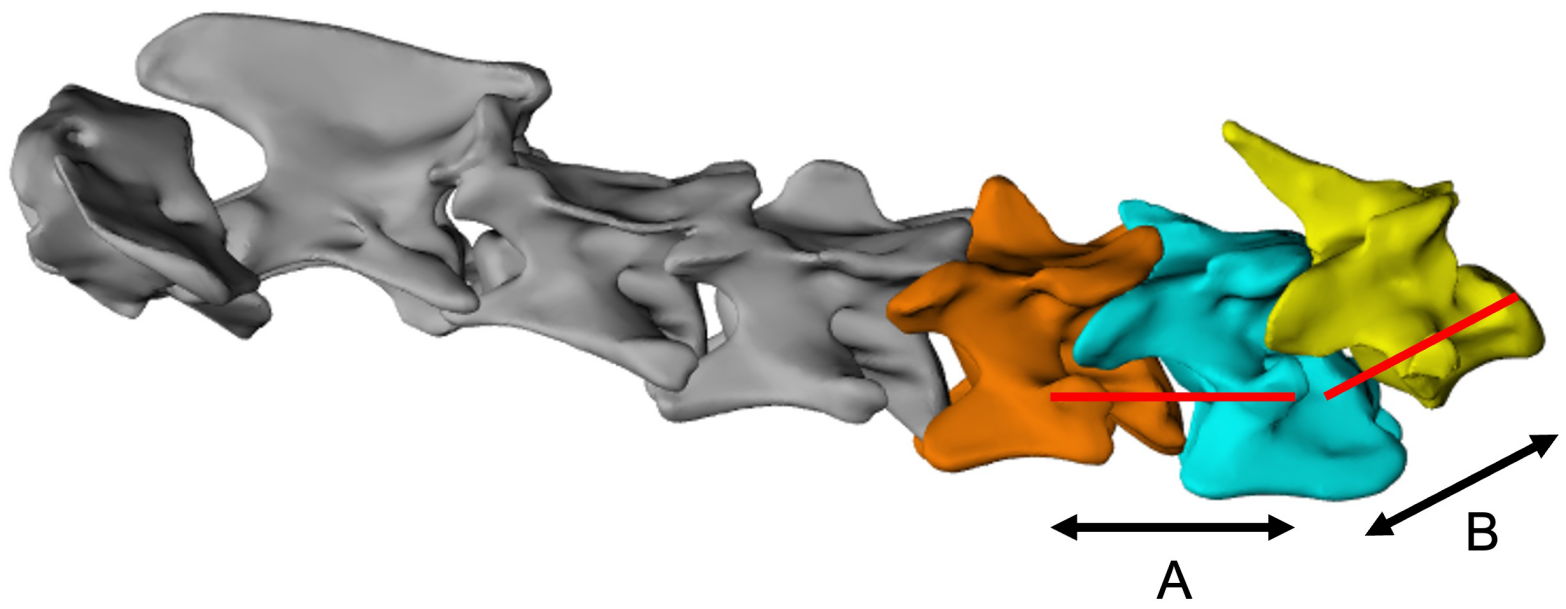
Virtual distraction of individual bone models using the sagittal axis of the corresponding caudal vertebra as a reference for each intervertebral disk. **(A)**, distraction axis for C5-C6; **(B)**, distraction axis for C6-C7.

The CT images were imported into Mimics software (Materialize NV, Leuven, Belgium) for the creation of individual 3D reconstructions of the affected vertebrae with the remaining cervical vertebra as a single model. These models were then exported into 3-matic software (Materialize NV, Leuven, Belgium) for further modeling, maintaining the desired orientation of vertebral lordosis from the pre-operative scans. Distraction was considered desirable to restore normal vertebral canal dimensions at affected levels, so a systematic approach was established to define the desired distraction level of the affected sites. The average value of non-affected cervical intervertebral disk ‘distances’ (i.e., the distance between two end-plates) were calculated and defined as the reference distance (i.e., with 0% distraction). The affected segments were then virtually distracted by the reference distance +10% using the sagittal axis of the caudal vertebra as a reference (Figure 1).

**Figure 2:**
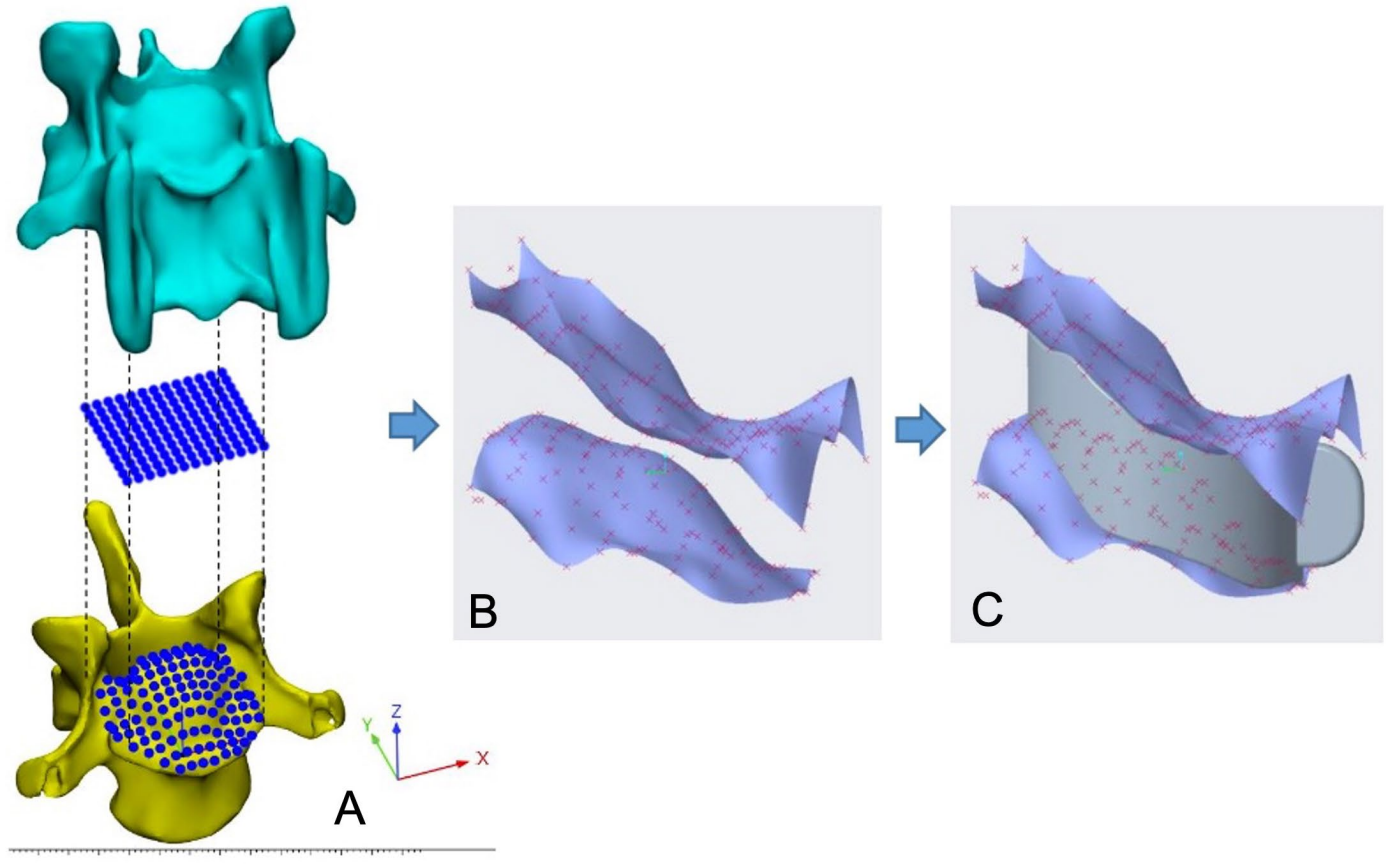
Mapping of endplate morphology data using the computer software ‘Parametric creo’. **(A)**, positioning of reference points; **(B)**, CAD of endplate surfaces; **(C)**, Endplate-conforming interbody spacer.

**Figure 3:**
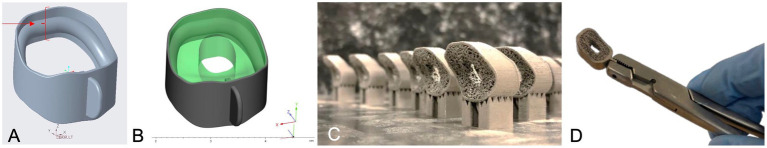
Interbody device design. **(A)**, ventrolateral view of solid section of device with distraction level arrowed; **(B)**, Solid part of spacer (in gray) and porous part of spacer (in green); **(C)**, image of selective laser melting build plate at manufacturing stage; **(C)** final device design being manipulated by ventral fin.

Once distracted at the intended fixation position, the vertebral end-plate morphology was determined using a 2-dimensional grid of reference points which was exported to Creo Parametric software (PTC, United States) for reference point blending and implant design (Figure 2). It was also considered desirable for the interbody device to encourage osseous integration with a porous structure. This was incorporated to each device using a technology previously reported to optimize bone in-growth (44–46). The device was completed with solid outer wall ‘ring’ to protect its structure, a central hole to accept autograft and a ventral ‘fin’ to assist implantation (Figure 3). The device was designed to measure 50% of the lateral width of the vertebral end-plate and 80% of the end-plate ventrodorsal height; these dimensions represented a compromise between end-plate coverage and ease of surgical implantation.

The interbody device was manufactured in titanium alloy (Ti6Al4V) using selective laser melting (SLM) system RebAM 500Q (Renishaw plc, Wooton-under-Edge, United Kingdom; Figure 3). The outer section was gently polished for a better surface finish, which did not compromise the bone-implant conformance. The devices were heat-treated, ultrasonically cleaned in distilled water for 40 min, dried at 120°C for 4 h and stored prior to surgical sterilization in steam at 121°C for 30 min.

Where applicable, vertebral models and surgical drill guides were designed and printed in polymer photocurable resin using stereolithography (Form 2, Formlabs; Somerville, United States) similar to methods described elsewhere (47).

### 2.4. Surgical procedure

Dogs were premedicated with 0.2–0.3 mg/kg intravenous or intramuscular methadone (Comfortan® 10 mg/mL, solution for injection for Dogs and Cats, Eurovet Animal Health B.V). General anesthesia was induced with propofol (Lipuro-Vet® 10 mg/mL solution for injection, Zoetis United Kingdom Ltd., UK) 4–6 mg/kg to effect and maintained with isoflurane (Iso-Vet® 1,000 mg/g inhalation vapor, Chanelle Pharma, Ireland). Analgesia was provided with continuous infusions of Ketamine (Narketan® 100 mg/mL solution for injection, Vetoquinol UK Ltd., United Kingdom) and Fentanyl (Fentadon® 50mcg/ml solution for injection, Dechra, UK). Cefuroxime (Zinacef GSK® Glaxo Operations UK Ltd., United Kingdom) was administered every 90 min during the procedure.

**Figure 4:**
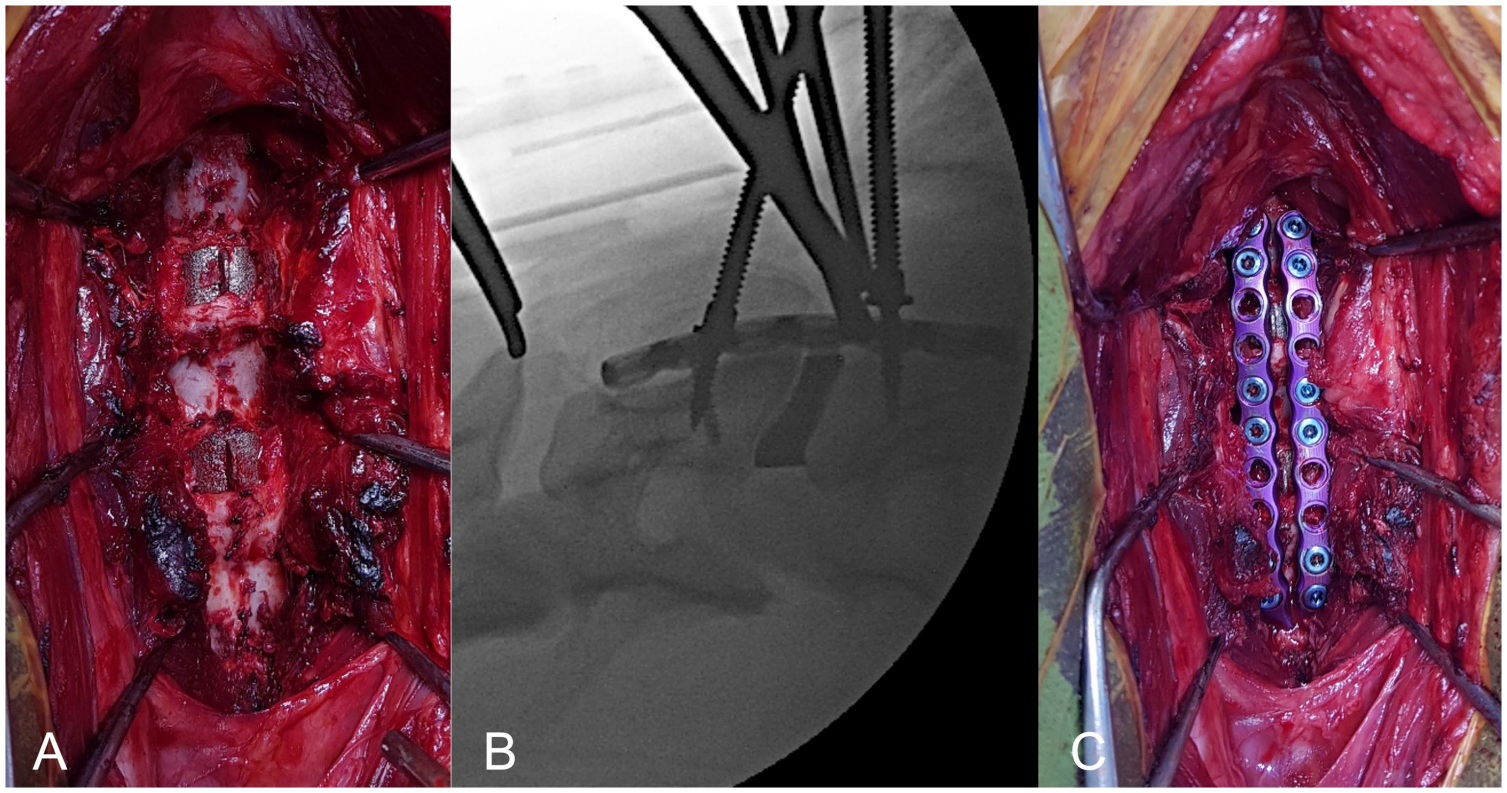
Surgical technique, ventral view of cervical spine. **(A)**, intervertebral spacers can be seen *in situ* (arrowed); **(B)**, intra-operative fluoroscopic image demonstrating adequate positioning of interbody spacer; **(C)**, pre-contoured paired ventral locking plates applied to vertebrae with mono-cortical locking screws.

Dogs were positioned in dorsal recumbency in a position as close to pre-operative imaging as possible. A standard ventral approach to the caudal cervical vertebra for exposure of the affected disk(s) and adjacent vertebral bodies was made. Sub-total discectomy was performed, preserving fibers of the dorsal and lateral annulus fibrosus. A self-retaining Caspar vertebral distractor (Aesculap, B Braun, Germany) was used to facilitate completion of the discectomy and for gentle curettage of the cartilaginous end-plates. In one case, a ventral slot of the adjacent end-plates was completed to penetrate the vertebral canal and remove compressive elements of the annulus fibrosus. The interbody device was test-fitted into the disk space and manipulated using needle holders prior to release of the distractors. Intra-operative fluoroscopy was used to confirm adequate insertion depth and end-plate conformation (Figure 4). Prior to insertion, the spacer was packed with cancellous autograft was harvested from one (single segment fusion) or both (dual segment fusion) proximal humeri. Mono- or bi-cortical secondary spinal stabilization was then performed using a method according to the preference of the surgeon using pre-operative *in-silico* planning, with or without the assistance of patient-specific 3D printed drill guides in the case of bi- and mono-cortical systems, respectively. Where possible these implants were also surrounded by cancellous autograft prior to routine closure.

Post-operative care included continuation of analgesic infusions for 24 h then intravenous or intramuscular methadone according to pain scores. Oral caprofen 2 mg/kg twice daily (Carprieve®, Norbrook, Ireland) or meloxicam 0.1 mg/kg once daily (Loxicom®, Norbrook, Ireland), paracetamol 10–15 mg/kg twice daily and gabapentin 10 mg/kg three times daily (Gabapentin Noumed®, Noumed, Life sciences Ltd., United Kingdom) was also administered. Antibiotics were not continued post-operatively. Post-operative recommendations included 3 weeks of strict rest with slowly increasing duration of controlled lead walks over the following six to 9 weeks according to the dogs ability.

### 2.5. Post-operative imaging

CT was performed immediately post-operative and at least at medium-term follow-up, with the patient positioned as per pre-operative scans. Scans were exported and multi-planar reconstructions were reviewed using open-source DICOM (digital imaging and communications in medicine) viewing software (Osirix)^1^ for implant stability, vertebral interbody fusion, distraction, and subsidence.

Interbody fusion was assessed in sagittally reconstructed images, aligned along the long axis of the vertebral body and interbody spacer. An ovoid region of interest (ROI) of approximately 5mm^2^ was created within the center of the interbody device and fusion was considered successful when the mean Hounsfield units (HU) for endoprosthetic bone density was between 266 and 1988.

Vertebral distraction and subsidence was determined using a measurement of the distance between the cranial and caudal vertebral end-plates of the segment (48), and a distraction index %, the method for which has previously been described utilizing the vertebral end-plates on lateral radiographs (29) and adapted here with determination of measurements taken from sagittally reconstructed CT scans. The index provides an assessment of end-plate measurements relative to individual vertebral morphometry. The end-plate measurements were calculated pre- and post-operative, then at medium-term follow-up; the indices were calculated immediately post-operative and then at medium-term follow-up. Subsidence was defined by a negative distraction index at medium-term follow-up.

Minor complications were classified as those requiring no further surgical intervention, major complications were classified by those that did. These were described and tabulated.

Where a third or fourth CT scan was performed at a minimum of 6 months follow-up, an assessment for adjacent segment pathology (ASP) was also made. ASP was defined by clear intervertebral disk space narrowing, significant vertebral end-plate changes and/or the development of spondylosis deformans, that did not cause clinical signs; adjacent segment disease (ASD) was recorded as those that did cause clinical signs (28, 33).

### 2.6. Statistical analysis

Continuous data sets were assessed for normality graphically and using Shapiro–Wilk tests. Mean and standard deviation (SD) were reported for normally distributed data. Categorical data are described showing the count and percentage. Paired t-tests were used to compare measurements on the same segment at two time points. Independent t-tests were undertaken to compare mean measurements and indices across two groups. The association between two independent measurements, the variation in TSCIS and the follow up distraction index, were assessed using Pearson’s correlation coefficient (r).

## 3. Results

### 3.1. Demographics and diagnosis

Nine medium and large breed dogs were included in this study, with mean age of 83.7 ± 19.8 months and weight of 42.7 ± 9.5 kg (Table 1). Breeds represented included three Doberman pinchers, two Great Danes, two Bernese mountain dogs, one Labrador Retriever and one border collie. There were 5 (55.6%) males and 4 (44.4%) females. All dogs were treated with analgesic medications and rest for a median period of 4 weeks (range 3–12) before non-surgical management was considered to have failed due to progression of clinical signs. All dogs were ambulatory at presentation, with typical clinical signs for DA-CSM including mild to moderate tetraparesis and generalized proprioceptive ataxia. The mean pre-operative TSCIS was 34.6 ± 1.98.

**Table 1:** Signalment, diagnosis and neurologic scores (FN, Female Neutered; TSCIS, Texas Spinal Cord Injury Score, ME, male entire; MN, male neutered).

						TSCIS	
Case	Breed	Age, m	Sex	Weight (Kg)	Affected level(s)	Pre-operative	Medium-term follow-up
1	Border Collie	104	MN	28.4	C6-C7	34	35
2	Doberman	123	FN	34.4	C5-C6, C6-C7	36	36
3	Labrador Retriever	108	MN	42.0	C6-C7	32	34
4	Great Dane	72	FN	38.5	C5-C6, C6-C7	32	35
5	Great Dane	70	FN	57.6	C6-C7	33	37
6	Doberman	72	MN	41.6	C5-C6, C6-C7	37	40
7	Bernese mountain Dog	60	MN	56.9	C5-C6	36	32
8	Doberman	84	ME	41.7	C5-C6, C6-C7	36	40
9	Bernese mountain dog	80	FN	52.9	C6-C7	33	40

All cases were diagnosed on MRI by the demonstration of focal intra-parenchymal spinal cord lesions in the C5-C7 region at the level of extra-dural cord compression and concurrent degenerative changes in the intervertebral disk, articular facet joint and interarcuate ligaments. Five dogs were diagnosed with DA-CSM at one segment (C6-C7 in four cases, C5-6 in one) and four dogs were diagnosed with DA-CSM at two segments (C5-C6 and C6-C7), totaling 13 operated segments.

### 3.2. Surgery

**Figure 5:**
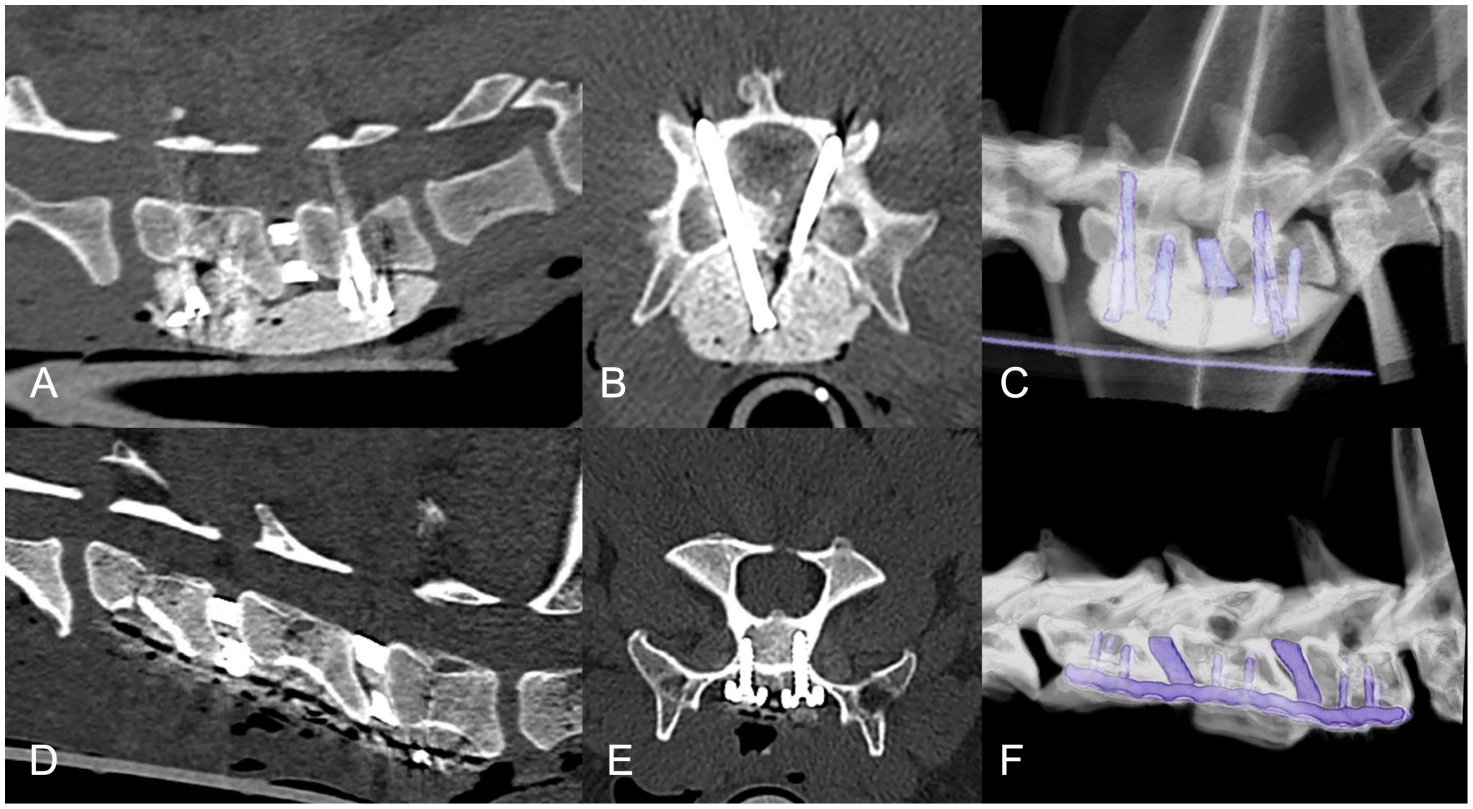
Post-operative CT imaging in cases with bi-cortical **(A–C)** and mono-cortical **(D–F)** supporting stabilization systems. Sagittal **(A,D)**, transverse **(B,E)** and three-dimensional **(C,F)** reconstructions with a bone algorithm.

All 13 interbody devices were inserted as planned (Figure 5). As previously described, one dog had a small ventral slot procedure contemporaneous with implantation of the interbody device. The supporting stabilization system utilized bi-cortical implants at 7 segments (in 5 cases); 5 segments (4 cases) with paired 2.7 or 3.5 mm bi-cortical locking screws with a single mono-cortical screw (DuPuy Synthes, Johnson and Johnson, United States) per vertebra which were enshrouded in a sculpted bolus of gentamicin-impregnated bone cement (Eurofix®, Synimed, France); and 2 segments (1 case) with a 3.5 mm pedicle screw and 3.2 mm rod system (Artemis®, Veterinary Orthopedics, United Kingdom). For the remaining 6 segments (4 cases), paired 8, 10 or 12 hole 2.7 mm locking plates and mono-cortical locking screws (Eickloxx, Eickemeyer Veterinary Equipment Ltd., United Kingdom) were utilized (Table 2). All bi-cortical implants were placed with the assistance of patient-specific 3D printed drill guides, whereas mono-cortical implants were placed without additional guidance. There were no notable challenges in the surgical procedure or patient anesthesia.

**Table 2:** Surgical technique, radiographic follow-up and long-term outcome (ASP, adjacent segment pathology; F/U, follow-up).

			End-plate measurements (mm)			Distraction index %		Medium-term CT findings			Long-term CT findings	Long-term outcome		
Case	Affected segment	Supportive stabilization	Pre-op	Post-op	F/U	Post-op	F/U	Subsidence	Fusion	Minor complications	ASP	F/U (months)	Outcome	Cause of euthanasia
1	C6-C7	Screws + bone cement	43.6	45.2	42.5	3.67	−2.52	Yes	Yes	Fractured bone cement	-	10	Improved	-
2	C5-C6	Screws + bone cement	52.83	55.1	54.5	4.30	3.16	No	No	None	-	12	Improved	-
	C6-C7	Screws + bone cement	48.54	49.33	49.23	1.63	1.42	No	Yes	Broken screw in C7	-			-
3	C6-C7	Screws + bone cement	47.98	50.53	49.39	5.31	2.94	No	Yes	None	No	9	Improved	-
4	C5-C6	Paired locking plates	56.46	59.35	57.85	5.12	2.46	No	Yes	None	No	24	Improved	Neoplasia
	C6-C7	Paired locking plates	49.2	50.58	49.21	2.80	0.02	No	Yes	1/12 screws backed out of plate in C7, 2/12 loose	Yes			
5	C6-C7	Paired locking plates	72.03	73.01	72.1	1.36	0.10	No	Yes	2/12 screws backed out of plate in C7	Yes	33	Improved	-
6	C5-C6	Paired locking plates	59.21	60.68	60.55	2.48	2.26	No	Yes	None	No	20	Improved	-
	C6-C7	Paired locking plates	55.77	55.9	54.33	0.23	−2.58	Yes	Yes	4/12 screws backed out of plate in C7	No			-
7	C5-C6	Paired locking plates	58.97	59.72	54.92	1.27	−6.87	Yes*	Yes	1/8 screw backed out of plate in C7, 1/8 loose	-	9	Deteriorated	IMPA
8	C5-C6	Pedicle screws - rod	61.3	61.6	61.2	0.49	0.16	No	Yes	Minor vertebral canal breach	Yes	13	Improved	Heart Failure
	C6-C7	Pedicle screws - rod	53.1	55.1	54.5	3.77	2.64	No	Yes	None	Yes			
9	C6-C7	Screws + bone cement	53.8	55.2	53.9	2.60	0.19	No	Yes	None	No	12	Improved	-

Asterisk denotes clinically relevant vertebral subsidence.

### 3.3. Clinical outcomes

The median duration of hospitalization prior to discharge was 5 days (range 3 to 7). In three dogs, transient worsening of neck pain and mild thoracic limb lameness was evident, which resolved during hospitalization. In the case that underwent a ventral slot, transient neurologic deterioration occurred which reversed to pre-operative function within 3 days.

The medium-term follow-up examination occurred from 2 to 8 months post-operative; mean 3.00 ± 1.82 months. At this point, 7 dogs had improved, 1 was stable and 1 had deteriorated with slight worsening of thoracic limb paresis and the development of pelvic limb lameness. This dog was subsequently diagnosed with immune-mediated polyarthritis (IMPA) following arthrocentesis of multiple joints. The mean TSCIS had improved significantly to 36.9 ± 2.78 (*p* = 0.009).

Long-term follow-up ranged from 9 to 33 months (mean duration 14.23 ± 8.24 months). Improvement was seen in 8 of the 9 dogs. The dog that suffered worsened thoracic limb paresis and was diagnosed with IMPA was euthanased 9 months post-operative due to unacceptable side-effects of corticosteroid therapy (polydipsia and polyuria). One dog was euthanased at 24 months post-operative for non-specific neoplasia, and one at 13 months due to the development of heart failure. One was diagnosed with gastrointestinal lymphoma 27 months post-operative and was under care for this disease without neurologic change at the last follow-up at 33 months post-operative (Table 2).

### 3.4. Radiologic outcomes

CT scans were performed immediately post-operative and at medium-term follow-up in all cases; findings are summarized in Table 2.

**Figure 6:**
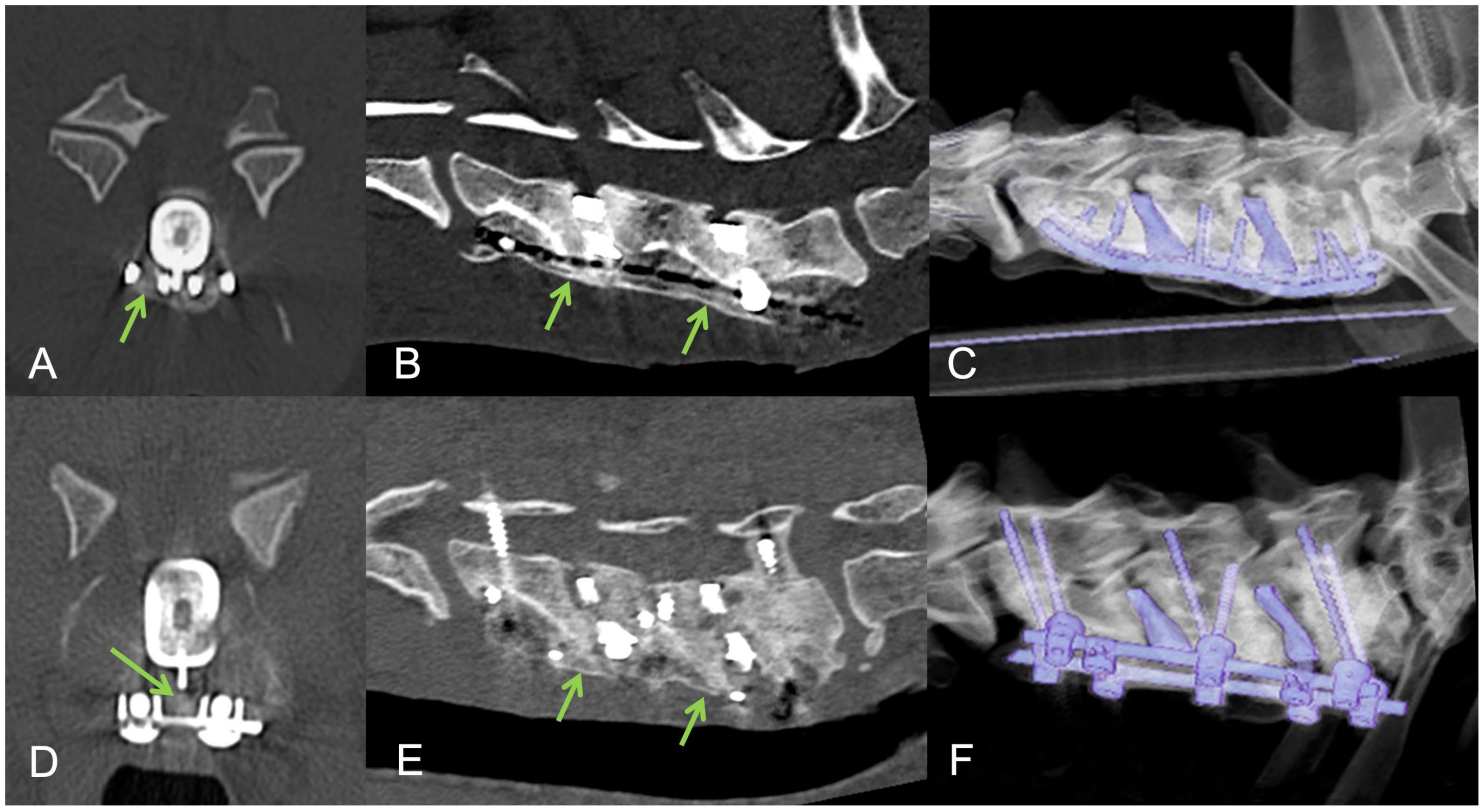
Follow-up CT examinations in cases with mono-cortical **(A–C)** and bi-cortical **(D–F)** supporting stabilization systems. Transverse **(A,D)**, sagittal **(B,E)** and three-dimensional **(C,F)** reconstructions with a bone algorithm. Ventral bony bridging (arrowed) and endoprosthetic osseous fusion is suggested.

**Figure 7:**
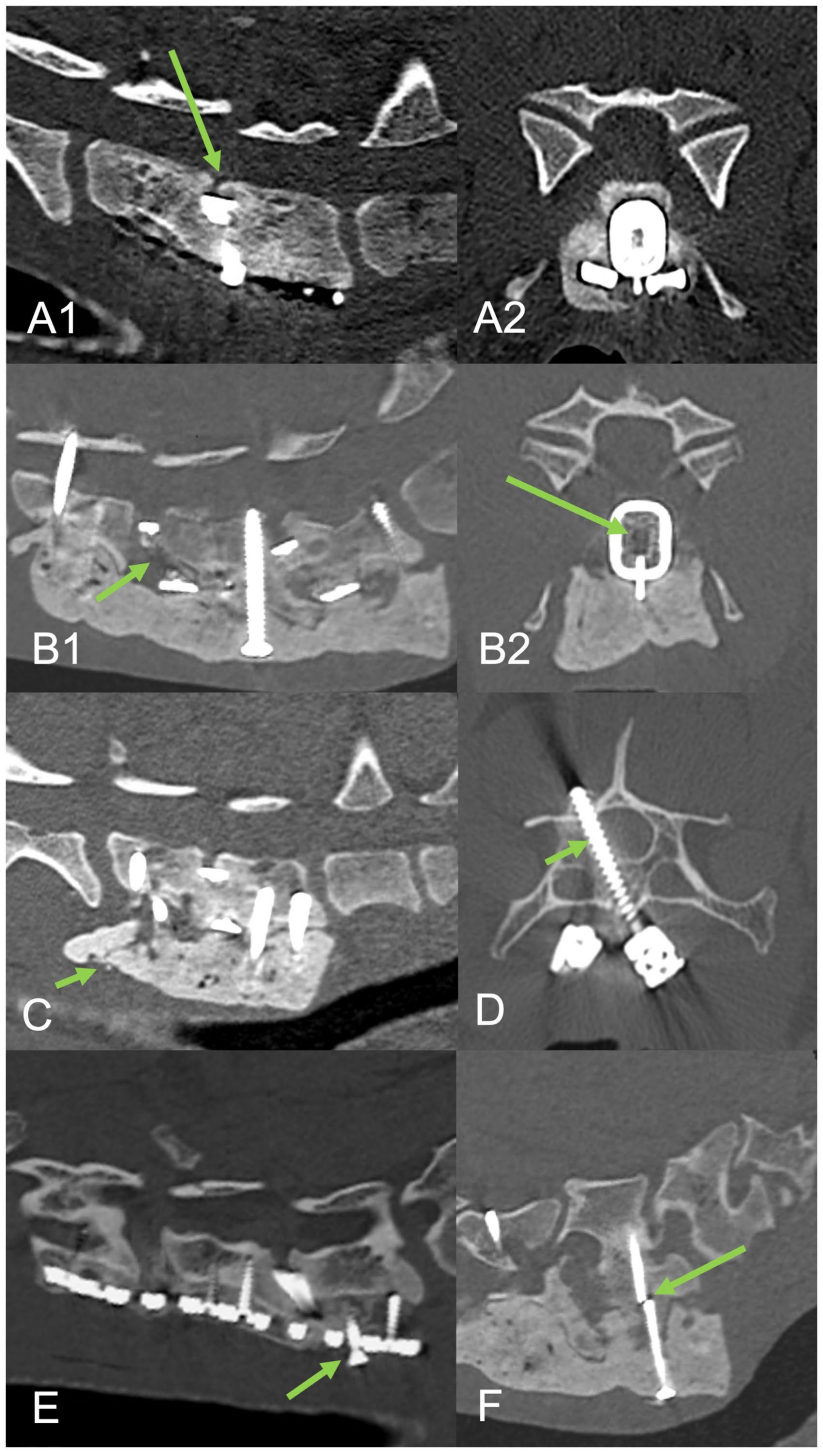
Complications associated with procedure. Top row sagittal **(A1)** and transverse **(A2)** CT reconstructions from case 7 that suffered mild neurologic deterioration associated with vertebral subsidence (arrowed) despite interbody fusion. Second row sagittal **(B1)** and transverse **(B2)** CT reconstructions from case 2 demonstrating a lack of interbody fusion at C5-C6 (arrowed) adjacent to a ventral slot. Other minor complications included cement fracture with mild subsidence **(C)** arrowed, mild vertebral canal screw breach **(D)** arrowed, screw back-out with mild subsidence **(E)** arrowed, and screw breakage **(F)** arrowed.

CT-determined interbody fusion was apparently successful in 8/9 cases (12/13 segments) (Figure 6); in the segment without fusion, endoprosthetic fusion appeared hampered by the ventral slot procedure performed (Figure 7). As locking screws and bone cement were utilized for secondary stabilization in this case, fusion ventral to the bodies could also not be appreciated. Bony-bridging ventral to the vertebral bodies was appreciated in all cases where bone cement was not utilized.

Minor complications were evident in 7/9 cases at 7/13 segments (Figure 7). These included one screw breakage, one fractured bone cement, two partial vertebral canal breaches and four partial back-out of locking screws from the caudal aspect of the locking plates, all of which occurred within C7. There were no major complications. Dislodging, displacement or failure of the interbody device was not seen at any segment.

Mean end-plate measurements for pre-, post-operative and medium-term follow up were 54.83 ± 7.25 mm, 56.25 ± 7.01 mm and 54.95 ± 7.23 mm, respectively. There was a statistically significant difference between pre- and post-operative measurements (*p* < 0.001) but also comparing post-operative to follow-up measurements (*p* = 0.003). Follow-up measurements did not vary significantly to pre-op (*p* = 0.787). The distraction index increased in all cases immediately post-operative (2.69 ± 1.67%) then reduced at follow-up in all segments (0.24 ± 2.86%), but remained positive in all but three segments (Table 2). Subsidence was therefore evident in 3/9 cases at 3/13 segments. At two segments, this subsidence appeared mild, but in one case subsidence appeared significant (distraction index −6.87%) and was also considered to be clinically relevant (Figure 7). There was no correlation between the variation of TSCIS pre- and post-operative and the distraction index at follow up (*r* = 0.3, *p* = 0.328).

Five cases returned for a fourth CT scan between 6 and 12 months post-operative. In one case, the medium-term follow-up scan occurred at 8 months, meaning a total of 6/9 cases had scans >6 months post-operative available for assessment of ASP at 10 segments. ASP was evident at 4/9 segments, all of which involved partial bridging spondylosis deformans at the adjacent disk space. ASP did not cause clinically relevant ASD in the study subjects.

## 4. Discussion

Patient specific endplate-conforming interbody devices with a micro-porous structure were developed and successfully implanted in nine dogs with DA-CSM alongside bi- or mono-cortical instrumentation and led to CT-determined fusion in 12 of 13 operated segments. There was a positive clinical benefit in 8 of 9 dogs and revision surgery was not required; the dog that demonstrated mild clinical deterioration might have maintained an acceptable level of function and quality of life or indeed improved, in advance of the natural progression of the disease, were it not for the development of co-morbid IMPA which affected mobility in the follow-up period. Our results compare favorably to the literature where success rates of 70–90% for surgical management of DA-CSM are reported (9, 11–17) and the endplate confirming nature of the implant limited clinically relevant subsidence as has been reported with other interbody devices previously (26–35).

This study represents the first description of an endplate conforming interbody device that was manufactured in titanium alloy by SLM with a micro-porous structure. Titanium alloy is an ideal material for this purpose given its rigidity, biological compatibility, corrosion resistance and MRI compatibility (49–52). Scaffold properties, such as porosity, porous size, permeability, stiffness and geometry can influence the success of bone in-growth (44, 45). The internal portion of the current device features a scaffold with properties that closely mimics trabecular bone (46) and which has already been successfully used in canine orthopedic surgeries including tibial tuberosity advancement osteotomies. A potential disadvantage for patient specific devices is the time needed to design and manufacture, meaning they are potentially not suitable for non-ambulant or emergency cases requiring more urgent surgery on a welfare basis; however, this is often not the case with DA-CSM.

Generic canine (31, 34) and human (33) interbody spacers have been successfully employed in dogs with DA-CSM, however, the canine interbody space is geometrically more complex than in humans, and simple convex or wedge-shaped cages will not perfectly conform. This limits bone-implant contact and load distribution, which may provide a less favorable biomechanical environment compromising endplate integrity leading to implant subsidence (42). Further, higher shear forces are present in the canine caudal cervical spine due to their naturally lordotic posture which can influence implant failure or migration (35, 41). The current interbody device was created with a high-friction surface with protective solid outer wall structure, which provided excellent end-plate conformity; implant failure, migration or infection was not seen in any case.

Implant subsidence was not completely avoided, with 3/13 operated segments having a reduction in distraction index relative to pre-operative levels. This was only clinically relevant in one case with one operated segment; however, revision surgery was not considered necessary in this case as the patient remained ambulant with a relatively mild change in gait. These results compare favorably with the current literature regarding interbody device subsidence (26–35), but direct comparison cannot be made on the basis of this study. There are significant variations in the methodology for assessing implant subsidence. We chose to assess subsidence using previous methodology (29, 33) adapted from human medicine (48) but applied to CT in follow-up. While some variation in patient positioning can be expected, the use of CT (where multi-planar reconstruction allows the fused segment to be assessed in an optimized mid-sagittal alignment) provided a potentially more accurate critique of the technique than would be possible with follow-up radiography only. Inter-observer agreement could be a source of future study to further validate these techniques.

Restoration of vertebral canal dimensions following intervertebral disk narrowing and protrusion is one of the main aims of managing DA-CSM (1, 2). Vertebral distraction can be beneficial in immediately decompressing the spinal cord and nerve roots (29) but over-distraction has been associated with pain in humans (53) and could in theory increase the risk of implant subsidence. A systematic approach to determining vertebral distraction was created in the current study, allowing for a marginal degree of distraction (+10%) from averaged non-affected disk spaces. This facilitated both ease of surgical implantation and an increased post-operative distraction index in all cases. This was well tolerated with hospitalization periods being less than a week; however, 3 cases demonstrated a transient worsening of lameness which could have related to one or multiple factors including over-distraction, surgical irritation of the nerve roots or the collection of autografts from the proximal humeri.

Supporting or ‘secondary’ stabilization systems are commonly employed in canine cervical interbody fusion studies (28–35), primarily citing the improved rate of interbody fusion with combined cage distraction and anterior plating in man (39). Mono-cortical systems such as paired locking plates are most often utilized (22–25, 28–30, 33, 35) as the relative risk of vertebral canal penetration is low and they can provide adequate stability after ventral slot procedures (54). Minor complications such as screw breakage and plate back-out are relatively common, as was the case in the current study (4/6 operated segments). Fortunately, implant loosening might be less significant when stabilization from fusion or bone bridging has already been accomplished (33). The caudal cervical spine experiences three times more axial rotation than the cranial cervical region (6) which may relate to more concave articular facet morphology in this region (55). Biomechanical assessment of the most caudal segments following mono-cortical stabilization or complete or sub-total discectomy is lacking. Some authors have proposed a combined dorsal and ventral approach to include trans-articular stabilization (30, 35) or a modified slot technique with bi-cortical transpedicular screws placed with the assistance of patient-specific 3D printed drill guides (56). In the current study, the choice of secondary stabilization system for each case was based on implant availability, implant cost and surgeon preference. We used guides for the placement of bi-cortical screws in combination with bone cement or rods in 7/13 segments and 3/7 experienced mild complications. The guided placement of pedicle screws with rods combines the benefits of bi-cortical fixation and the ability to lay down large amounts of cancellous autograft which assists with ventral bony bridging and potentially more rapid fusion. This technique is now being utilized in the authors institution and warrants further study.

A significant limitation to the current study is the lack of histologic documentation of interbody fusion post-mortem, which might have been possible from one of the euthanased cases. Unfortunately, the bodies were not made available for study which was primarily related to distance of travel and euthanasia in a primary care setting. In addition, the determination of fusion was based on CT, rather than more sophisticated modalities such as μCT or *in-silico* subtraction techniques to reduce metallic scatter (33). Ideally, a calibration phantom would be used as a control (56). Despite this limitation, interbody fusion was considered very likely in 12/13 segments based on the ROI Hounsfield units being compatible with bone. It remains possible that individual segments lacked complete compound bone fusion and/or contained fibrous elements within the interbody spacers.

The assessment of ASP was possible in 6/9 cases, revealing 3 cases with 4/9 segments affected; fortunately, clinical signs suggestive of ASD were not reported in any cases on long-term follow up. Distraction-fusion techniques are expected to alter vertebral motion patterns. It is currently unknown whether endplate conforming interbody devices will reduce the risk of ASP and ASD with time, relative to non-conforming spacers or cages. Current devices developed to maintain normal motion and reduce ASD, such as prosthetic disks, may be more prone to failure than distraction-fusion techniques (35). ASP can be influenced by plates and screws impinging on adjacent segments (33), which was avoided in this case series. The complete assessment for ASP and ASD with our technique is hampered by the retrospective nature of this study and a lack of uniform long-term follow up.

Other potential limitations to this study include its descriptive nature, small sample size, potential selection bias (based on financial viability and/or the selection of dogs with only mild to moderate clinical signs) and the lack of a control group. Direct comparisons to other interbody devices/spacers/cages are not possible. Further biomechanical and clinical studies would be required in dogs to confirm the suggestion that the devices provide a superior environment for stability and osseous in-growth.

In conclusion, we reported the successful development and application of patient specific, endplate conforming interbody devices, featuring a micro-porous structure, for the instrumented distraction and fusion of 13 diseased spinal segments in nine dogs with DA-CSM. The technique was safe and effective, with no significant intra-operative complications. There were only minor implant-related complications on follow-up, none of which required revision surgery; in one case, subsidence was associated with mild clinical deterioration, the remaining eight cases improved.

## Data availability statement

The raw data supporting the conclusions of this article will be made available by the authors, without undue reservation.

## Ethics statement

The animal study was reviewed and approved by CVS Group Internal Ethical review commitee. Written informed consent was obtained from the owners for the participation of their animals in this study.

## Author contributions

CD, JR, VL, and DJ gave an essential contribution to the design and development of the implants. BW assisted in refinement of the system. VL performed the *in-silico* modeling and implant manufacture. CD, JR, RF, and AT managed and operated the clinical cases, collected data and contributed to the writing of the article. All authors contributed to the article and approved the submitted version.

## Conflict of interest

BW and DJ are directors of Fusion Implants Ltd. who assisted the design of and manufactured the interbody devices.

The remaining authors declare that the research was conducted in the absence of any commercial or financial relationships that could be construed as a potential conflict of interest.

## Publisher’s note

All claims expressed in this article are solely those of the authors and do not necessarily represent those of their affiliated organizations, or those of the publisher, the editors and the reviewers. Any product that may be evaluated in this article, or claim that may be made by its manufacturer, is not guaranteed or endorsed by the publisher.

## Footnotes

^1^ www.osirixviewer.com
